# Supplementary material for: Multi-subunit collaboration enables Smc5/6 to function as a composite SUMO E3 complex
Source: Nat Commun. 2026 Jul 23;17:8969. doi: 10.1038/s41467-026-75592-7 (PMC13503820; doi:10.1038/s41467-026-75592-7)
Supplement: Supplementary file 3 [file 41467_2026_75592_MOESM3_ESM.pdf]

nature portfolio

# Peer Review File

# Multi-subunit collaboration enables Smc5/6 to function as a composite SUMO E3 complex

Corresponding Author: Dr Xiaolan Zhao

**This file contains all reviewer reports in order by version, followed by all author rebuttals in order by version.**

Version 0:

Reviewer comments:

Reviewer #1

(Remarks to the Author)
The authors of the manuscript „ Multi-subunit collaboration enables Smc5/6 to act as a composite SUMO E3 complex " show reduced levels of SUMOylation of Smc5, Smc6, and Sgs1 substrates upon depletion of SMC5/6 subunits. Then, they purified SMC5/6 core and holo-complexes and used them for an in vitro SUMOylation assay. The binding of ATP and DNA to the purified complexes stimulated the SUMOylation, suggesting that the whole complex and its dynamics modulate Nse2 ligase activity. Particularly, the ATP-bound ring-like configuration supported SUMOylation, suggesting that a Smc6-free Smc5 arm favours Nse2 activity. Although the study provides another step in characterizing Nse2 SUMO ligase activity, I have several reservations:

Major:
1. The authors build on previous findings that the intact Smc5/6 complex is required for the activity of its associated Nse2 SUMO ligase (Bermudez-Lopez et al., PLoS Biol, 2015). Here, they depleted Smc5, Smc6, Nse4, Nse5 or Nse6 subunits, which had an indirect effect on SUMOylation levels. To convince readers that these SUMO modifications are imposed by Nse2, an essential control showing reduced SUMO levels upon Nse2 depletion should be included.

2. In Fig. 2, it is shown that the Nse2 ligase activity is higher in complex with (only) Smc5 compared to core- or holo-complexes. However, direct comparisons of Nse2-Smc5 dimer to DNA-bound complexes are missing (Figs. 3 and following). As Varejao et al., (EMBO J, 2018) have already shown stimulation of SUMOylation through the DNA binding to Smc5 arm, these comparisons should be added and discussed.

3. Different DNA-binding mutations exhibit different levels of SUMOylation, and the authors suggest that this is due to their different effects on association with DNA. However, they do not provide any EMSA (or other) data supporting these assumptions.

Minor:
1. The fact that the DNA binding to the Smc5 arm stimulates SUMOylation is ignored. The previous results by Varejao et al., (EMBO J, 2018) should be properly discussed.

2. It is hard to judge how much indirect (or direct) the effect of subunit depletion on SUMOylation in cells is. For example, if the depletion affects only Nse2 activity, one could expect stimulation upon Smc6 depletion as the Smc5 arm would be free (as in ring-like conformation or Smc5-Nse2 dimer). Could the authors discuss their results more thoroughly or conduct further experiments to address this issue?

3. In Suppl. Fig. 1c, "momo-SUMO form" should be corrected and SUMO-His8 should be used (instead of a small superscript).

Reviewer #2

(Remarks to the Author)

The authors present a series of (predominantly) biochemical experiments that use defined recombinant yeast Smc5/6 complexes in a reconstituted yeast SUMOylation system, showing that the complex is capable of auto-modification, as well as modification of Sgs1(BLM). A series of experiments that examine the effects of different DNA substrates on SUMOylation activity are also included. As it stands, the manuscript only offers an incremental increase in our understanding of how the Smc5/6 complex operates, with many of the experiments being confirmatory in nature, having been reported elsewhere. The authors also report a lot of "moderate" or "mild" effects, but do not offer definite results or conclusions.

MAJOR POINTS

Page 4, lines 139-144
The authors report that acute depletion of Smc6, Nse4 or Nse6 leads to a reduction in SUMOylation of Smc5 - and that depletion of Smc5 (and thus Nse2), Nse4, and Nse6 lead to a reduction in SUMOylation of Smc6. Given the interdependence of the complex on each of its constituent components, the only real insight offered here, is the potential effect of depleting Nse6 on the SUMOylation pathway [but see other comments below].

Page 5, lines 197-204 + Page 6, lines 205-211
"This is exemplified by Sgs1, the sumoylation of which is enhanced by its association with DNA repair intermediates"
"These observations let us ask whether non-SUMO E3 subunits of Smc5/6 can contribute to sumoylation in the presence of DNA"
"Smc5/6 can facilitate HJ processing partly by promoting Sgs1 sumoylation and preferentially binds to ds-ssDNA junctions and supercoiled DNA over dsDNA and ssDNA".

Given the statements above and the cited references, it is not clear what insight the authors are providing, over and above what has already been published. Furthermore, it is already known that the SUMOylation activity of Nse2 is stimulated by the presence of single-stranded DNA [Varejão et al, EMBO J, 2018, Varejão et al, Nature Comms 2021]. How do the authors square their observations with the published literature?

The authors also do not measure any kinetics for their reconstituted SUMOylation assays, making it difficult to accurately compare the activity of the different reconstituted complexes. This is especially apparent when comparing the 6-subunit Smc5/6 complex to the 8-subunit complex (i.e. +/- Nse5/6), for example in Figure 2 at the 4-minute time point, there is no statistical difference between the two complexes; closer observation shows that a single outlier in both datasets serves to create the visual difference.

Page 6, lines 213-244
"We added each type of DNA at the same nucleotide concentration in sumoylation reactions containing core-Smc5/6"

The importance of determining kinetics is once again seen in the experiments presented in Figure 3; here the differences observed after 10 minutes incubation time between the different subunits could be considered negligible; all supplied substrates are stimulatory with respect to the "no DNA" control.

Also, it is imperative that experiments are carried out in conditions close to, or in excess of the Kd for the interaction of the complex with the provided substrate, to facilitate direct comparison.

Page 6, Lines 230-234
"In the presence of core-Smc5/6, HJ and ds-ssDNA led to increased Sgs1 sumoylation compared with no DNA control"

Presumably, Sgs1 is active in this assay? The authors should understand the contribution of the helicase's activity to the stimulation of SUMOylation, i.e. generation of a more favourable DNA substrate upon unwinding.

Page 6, lines 253-254
"A recent cryo-EM structure showed that a single core-Smc5/6 binds to 20-bp dsDNA. Thus, the minimal DNA length required for sumoylation stimulation raised the possibility that two core-Smc5/6 complexes may sumoylate each other in-trans, with one acting as the E3 and the other as the substrate, when both are bound to the same dsDNA molecule"

Others have shown that more than one Smc5/6 complex can bind to longer stretches of dsDNA, and that the loop extrusion behaviour of the complex requires a dimer; please take care to provide citations to previous work that provide the foundation to your study.

Page 8, line 305-212
"In each case, levels of sumoylated Smc5-Smc6 were moderately reduced compared with the wild-type core-Smc5/6 (Fig. 5b), as were sumoylated Sgs1 levels (Fig. 5c). The moderate effects seen for each mutant are likely because DNA binding sites on the other subunits enable partial DNA association. These results provide in vitro evidence that DNA binding via four non-SUMO E3 subunits contributes to DNA enhanced Smc5/6 SUMO E3 activity".

The authors shy away from the more obvious conclusion that the complexes containing mutations which affect dsDNA binding have very little effect on the SUMOylation activity of the Smc5/6 complex. They should test the effects of hinge mutations that disrupt the ability of the complex to interact with single-stranded DNA; this is especially important given the authors previous statement that Smc5/6 "preferentially binds to ds-ssDNA junctions"; and previous publications linking the SUMOylation activity of Nse2 to single-stranded DNA binding.

Furthermore, given that Nse2 serves to bridge the two arms of the Smc5/6 complex (see Hallett et al, CryoEM structure of the Smc5/6 holo-complex, 2002). The authors should consider that ATP-binding serves to disrupt this interface (I to O transition), allowing access of the Nse2 subunit to DNA substrates (ssDNA, ds-ssDNA) leading to stimulation of its activity.

Figure 3
Could the authors explain why there are more replicates for experiments with the Holliday Junction substrate. Ideally all experiments should have the same number, in order not to affect the numerical results of a two-tailed unpaired t-test; consider use of a Welch t-test, when comparing data with unequal sample sizes / variances.

MINOR

Consider altering sumoylation to SUMOylation throughout.

Given that Nse5/6 are not obligatory parts of the Smc5/6 complex in either S. pombe or in humans, the use of the term 'holo-complex' should be ideally be reserved for the 6-subunit complex, and not used for the 8-subunit complex.

The use of 'non-SUMO E3' subunits as a terminology is potentially confusing, especially as there is actually only one SUMO E3 subunit, i.e. Nse2.

Page 6, lines 236-244
"Significantly, the additional of holo-Smc5/6 led to poly- and multi-sumoylation species of Sgs1, the level of which was increased by inclusion of HJ DNA in the reactions".

How are these being enumerated? Can the authors distinguish between the different species?

Version 1:

Reviewer comments:

Reviewer #1

(Remarks to the Author)
The authors rewrote the article and significantly improved its quality. The revised version has a more logical sequence of results and clearer conclusions.

I have only a minor objection to the arrangement of panels in the Figures – it would be clearer to arrange panels A - B ... horizontally (side by side, not one above the other and then randomly below).

Reviewer #3

(Remarks to the Author)
The authors have included additional data, improved quantification, and expanded the discussion to address Reviewer #2's comments, and in doing so have strengthened both the clarity and overall presentation of the work.

That said, several important concerns raised by Reviewer #2 remain only partially addressed.

The main conclusions of the manuscript, that ATP binding and DNA stimulate Smc5/6-dependent SUMOylation, are already established in the literature. While the use of the full Smc5/6 complex provides a more physiological context and represents a clear technical advance, the conceptual novelty relative to previous studies remains somewhat limited. As it stands, the manuscript primarily refines existing models rather than extending significantly our understanding.

The most potentially novel aspect of the study is the reported difference in DNA substrate preference, namely that double-stranded DNA (dsDNA) appears more stimulatory than single-stranded DNA (ssDNA), this is in contrast to earlier work using the Nse2–Smc5 subcomplex. However, this important observation is not yet developed with sufficient depth. In this context, a key request from Reviewer #2, arguably the most important one, remains unaddressed. The reviewer asked testing hinge mutants that disrupt ssDNA binding in the SUMOylation assays. Given the well-established role of the Smc5/6 hinge in mediating ssDNA interactions, this experiment is essential to directly compare how dsDNA vs ssDNA binding contributes to SUMOylation activity in the context of the full complex.

Importantly, this analysis should not be limited to a single condition. A systematic comparison between hinge mutant and wild-type complexes across the full range of DNA substrates used in the study, including ssDNA, dsDNA, and DNA junctions, should be included.

In summary, the manuscript is technically improved and clearer following revision. However, directly addressing the role of hinge-mediated ssDNA binding through systematic analysis of hinge mutants across relevant DNA substrates and their

effect on SUMOylation is critical to elevate the conceptual impact of the work otherwise the novelty of the work remains rather limited over previous studies.

Version 2:

Reviewer comments:

Reviewer #3

(Remarks to the Author)
I thank the authors for their thorough and detailed rebuttal. The additional poly-dT ssDNA experiment is informative and goes a meaningful way toward addressing the central concern about ssDNA versus dsDNA stimulation, demonstrating that structure-free ssDNA fails to stimulate SUMOylation by the intact complex is a substantive result that supports their conclusions. I am also persuaded by the mechanistic contrasts drawn with the Nse2-Smc5 dimer studies, particularly the proximity-based model, the ATP-DNA engagement argument, and the basal suppression concept. Taken together, these represent genuine conceptual advances and I am satisfied that the manuscript merits publication.
That said, I maintain that the hinge mutant question has not been fully resolved, it has been sidestepped rather than answered. The argument that ssDNA-binding residues are structurally uncertain and that generating a clean mutant is beyond scope is not entirely satisfying. The poly-dT experiment provides indirect evidence, but a direct test of hinge-mediated ssDNA binding in the context of the full complex would have been the cleaner experiment. I would encourage the authors to acknowledge this limitation explicitly in the manuscript rather than presenting the ssDNA insensitivity conclusion with full confidence. I recommend acceptance subject to this minor textual revision.

**Open Access** This Peer Review File is licensed under a Creative Commons Attribution 4.0 International License, which permits use, sharing, adaptation, distribution and reproduction in any medium or format, as long as you give appropriate credit to the original author(s) and the source, provide a link to the Creative Commons license, and indicate if changes were made.
In cases where reviewers are anonymous, credit should be given to 'Anonymous Referee' and the source.
The images or other third party material in this Peer Review File are included in the article's Creative Commons license, unless indicated otherwise in a credit line to the material. If material is not included in the article's Creative Commons license and your intended use is not permitted by statutory regulation or exceeds the permitted use, you will need to obtain permission directly from the copyright holder.
To view a copy of this license, visit https://creativecommons.org/licenses/by/4.0/

**Point-By-Point Response to Reviewers' Comments**

We thank the reviewers for their careful evaluations of our manuscript and their constructive suggestions. As detailed below, we address the points raised by the reviewers with new data (six new figure panels) as well as improved data plots that show fold changes and p-values derived from the suggested statistical analyses. In addition, we provided better description of previous studies and the advances offered from this work, as well as thorough edits of the text to concisely describe our results. These changes have improved the clarity and rigor of our work. Our responses below and textual changes that address reviewers' critiques are colored blue.

**Reviewer #1.** The authors of the manuscript "Multi-subunit collaboration enables Smc5/6 to act as a composite SUMO E3 complex" show reduced levels of SUMOylation of Smc5, Smc6, and Sgs1 substrates upon depletion of SMC5/6 subunits. Then, they purified SMC5/6 core and holo-complexes and used them for an in vitro SUMOylation assay. The binding of ATP and DNA to the purified complexes stimulated the SUMOylation, suggesting that the whole complex and its dynamics modulate Nse2 ligase activity. Particularly, the ATP-bound ring-like configuration supported SUMOylation, suggesting that a Smc6-free Smc5 arm favours Nse2 activity. Although the study provides another step in characterizing Nse2 SUMO ligase activity, I have several reservations:

Major:
1. The authors build on previous findings that the intact Smc5/6 complex is required for the activity of its associated Nse2 SUMO ligase (Bermudez-Lopez et al., PLoS Biol, 2015). Here, they depleted Smc5, Smc6, Nse4, Nse5 or Nse6 subunits, which had an indirect effect on SUMOylation levels. To convince readers that these SUMO modifications are imposed by Nse2, an essential control showing reduced SUMO levels upon Nse2 depletion should be included.

Previous studies from us and others have shown that Nse2 E3 mutants reduce the SUMOylation levels of Sgs1, Smc5, and Smc6 in cells, and that Nse2 can SUMOylate these proteins *in vitro*[1-4]. These studies thus established that Nse2 mediates the SUMOylation of the three proteins examined in this work. We have made these points more clearly in the revision (Page 4, lines 129-135).

2. In Fig. 2, it is shown that the Nse2 ligase activity is higher in complex with (only) Smc5 compared to core- or holo-complexes. However, direct comparisons of Nse2-Smc5 dimer to DNA-bound complexes are missing (Figs. 3 and following). As Varejao et al., (EMBO J, 2018) have already shown stimulation of SUMOylation through the DNA binding to Smc5 arm, these comparisons should be added and discussed.

Thanks for this great suggestion. We now included new data to show that compared with the Nse2-Smc5 dimer, the SUMOylation activity of Smc5/6 is stimulated to a much greater degree by DNA (new Figures 2d, 2f; Pages 5-6, lines 193-217). We showed that while DNA addition led up to a 12-fold stimulation of SUMOylation when Smc5/6 was used as the E3, less than 2-fold stimulation by DNA was seen when Nse2-Smc5 was the E3. Taking consideration of data already included in the manuscript, which showed that Nse2-Smc5 exhibited higher activity than Smc5/6 without DNA, this result suggests that as a complex, Smc5/6 can minimize promiscuous SUMOylation in the absence of DNA and achieves better activity in the presence of DNA. We believe this is an important conclusion from this work, and we thank the reviewer for recommending the test.

In addressing a related comment from the reviewer (more below), we note that Nse2 is an obligatory subunit of Smc5/6 and co-purifies with all seven other subunits, and that there is no evidence for the presence of Nse2-Smc5 dimer in cells[1,5-9]. *In vitro*, Smc5/6 behaves differently from Nse2-Smc5, as exemplified by the results described above. In another example, Smc5/6's SUMOylation activity is stimulated more strongly by dsDNA than by ssDNA as we shown here (new Figures 2b, 2c), contrasting from the finding reported by Varejao et al. (2018) wherein ssDNA was found to be better than dsDNA in stimulating Nse2-Smc5's activity. These differences suggest that while previous studies of Nse2-Smc5 are valuable, conclusions drawn in those studies often do not apply to Smc5/6, likely because it contains 6 additional subunits that enable more intricate regulation of the complex function in SUMOylation. It is thus critical to examine Smc5/6 to derive how its E3 activity is regulated (See Introduction, Page 3-4, lines 97-106).

3. Different DNA-binding mutations exhibit different levels of SUMOylation, and the authors suggest that this is due to their different effects on association with DNA. However, they do not provide any EMSA (or other) data supporting these assumptions.

We now included the requested EMSA data for the mutant complexes. The four examined DNA-binding mutant complexes exhibited reduced DNA-binding ability (new Supp. Fig. S4b; Page 7, lines 278-281). We note that while DNA-binding mutant complexes reduced SUMOylation to varied degrees, the differences between the mutants were not statistically significant.

Minor:

1. The fact that the DNA binding to the Smc5 arm stimulates SUMOylation is ignored. The previous results by Varejao et al., (EMBO J, 2018) should be properly discussed.

We have now discussed several differences between the cited study and our work (Page 10, lines 441-446). First, as described above, the two studies using different E3 forms conclude contrarily about the effects of ssDNA versus dsDNA on SUMOylation. We note that while earlier work studied Nse2-Smc5 as Smc5/6 was difficult to purify, this dimer is not a physiological entity in the cells. Rather, Nse2 is an obligatory subunit of Smc5/6 and co-purifies with all seven subunits of Smc5/6[1,5-9]. Second, our work examining Smc5/6 in SUMOylation provides numerous new insights not offered in the study of Nse2-Smc5 by Varejao et al (2018). For example, we showed that dsDNA stimulates Smc5/6 SUMOylation activity via binding to four of its non-E3 subunits. Further, we revealed how ATP binding aids SUMOylation via conformational changes, which can only occur in the complete complex and not in Nse2-Smc5. Third, we provide three lines of evidence to support the conclusion that DNA promotes substrate and E3 proximity, which was not seen for Nse2-Smc5. With these differences noted, our data do not exclude a potential role of Smc5-arm in SUMOylation. In summary, our conclusions derived from examining Smc5/6 and its multiple constituents support a shared principle with multi-subunit UBL E3 complexes, which also utilize intricate collaboration among all non-E3 subunits to achieve modification efficiency and specificity[10-12].

2. It is hard to judge how much indirect (or direct) the effect of subunit depletion on SUMOylation in cells is. For example, if the depletion affects only Nse2 activity, one could expect stimulation upon Smc6 depletion as the Smc5 arm would be free (as in ring-like conformation or Smc5-Nse2 dimer). Could the authors discuss their results more thoroughly or conduct further experiments to address this issue?

We have added discussion on how loss of Smc5/6 subunits is likely to affect SUMOylation via multiple means in cells (Page 12, lines 525-529). Using Smc6 as an example, its loss not only

affects Smc5/6's conformation, but also its DNA binding and ATPase activities, since both require Smc6[8,9]. As such, acute depleting Smc6 can simultaneously alter the complex's structure and reduce its proximity with substrates on DNA for SUMOylation. Similarly, Smc5 and the Nse subunits have multiple structural and biochemical activities, thus they can also affect cellular SUMOylation in more than one way. While the full picture of how each subunit integrates its multiple biochemical activities in regulating SUMOylation awaits further study, our work provides a critical step toward this goal by unveiling how DNA binding by Smc5, Smc6, Nse3, and Nse4 and how ATP binding by Smc5 and Smc6 can contribute to SUMOylation.

3. In Suppl. Fig. 1c, "momo-SUMO form" should be corrected and SUMO-His8 should be used (instead of a small superscript).
The spelling errors are corrected in the said panel (now Suppl. Fig. S6e); thank you.

**Reviewer #2.** The authors present a series of (predominantly) biochemical experiments that use defined recombinant yeast Smc5/6 complexes in a reconstituted yeast SUMOylation system, showing that the complex is capable of auto-modification, as well as modification of Sgs1(BLM). A series of experiments that examine the effects of different DNA substrates on SUMOylation activity are also included. As it stands, the manuscript only offers an incremental increase in o ur understanding of how the Smc5/6 complex operates, with many of the experiments being confirmatory in nature, having been reported elsewhere. The authors also report a lot of "moderate" or "mild" effects, but do not offer definite results or conclusions.

Our work goes beyond confirming published data, and we report clear effects on the Smc5/6 E3 activity by DNA, ATP, and six types of mutants. Previously, biochemical studies examined the Nse2-Smc5 dimer in SUMOylation; however, the dimer form is not present in cells, nor can it support *in vitro* DNA- or ATP-binding activities exhibited by Smc5/6 without other subunits[1,5-9,13]. Differing from these studies, we address how the complete Smc5/6 complex acts in SUMOylation through its multiple subunits. Our data provide new perspectives from those derived from Nse2-Smc5 studies and support a shared principle with other multi-subunit UBL E3 complexes, which also utilize collaboration among all non-E3 subunits to achieve modification efficiency and specificity[10-12]. We summarize below our new findings and how they differ from previous ones. We have adjusted section titles and made textual changes to better highlight the advances made in this work.

1) ***Roles of Smc5/6 subunits not previously examined.*** Our *in vitro* and *in vivo* data demonstrate the roles of subunits not examined before in SUMOylation, including Nse3, Nse4, and Smc6. We show that their roles in SUMOylation are mediated by their abilities in binding to dsDNA and ATP. These findings highlight the importance of examining the complete complex rather than just Nse2-Smc5 to derive action mechanisms of Smc5/6.
2) ***SUMOylation of physiologic substrates.*** Previous studies of the SUMOylation mechanism of Nse2-Smc5 examined a fragment of the non-yeast protein p53 or a fragment of Nse4 as substrate [2,14,15]. In contrast, we examined physiological substrates, including the full-length Sgs1, Smc5, and Smc6, the SUMOylation of which has been shown to contribute to genome maintenance [1-4,16,17]. Our results thus provide an understanding of SUMOylation mechanisms in more physiological settings than the previous studies.
3) ***dsDNA is superior to ssDNA in stimulating Smc5/6-based SUMOylation.*** While a previous study of the Nse2-Smc5 dimer showed that ssDNA is superior to dsDNA in stimulating SUMOylation[14], we found the opposite for Smc5/6. For example, data in Figure 2b show that at a 5-min reaction time, dsDNA led to a 10-fold stimulation of SUMOylation

whereas ssDNA had less than a 2-fold stimulation. This difference highlights the distinct behaviors of Smc5/6 versus Nse2-Smc5 dimer in SUMOylation.

4) ***Mechanisms of dsDNA-based stimulation.*** We show that DNA stimulation of SUMOylation arises from promoting proximity between the SUMO E3 complex and the substrate. This insight was not provided in previous studies. Another novel finding in our work is that dsDNA-binding residues on Nse3, Nse4, Smc5, and Smc6 mapped by structural studies are shown to be involved in DNA-based SUMO E3 stimulation.

5) ***Roles of ATP binding in promoting SUMO E3 function.*** Our data reveal how ATP-binding can support the SUMO E3 activity of Smc5/6. This conclusion cannot be properly reached by examining Nse2-Smc5, since ATP binding by Smc5/6 can only be achieved when Smc5 and Smc6 both are present to sandwich ATP molecules between them[8,9]. We also provide structural basis to explain how ATP-induced structural changes of Smc5/6 allow Nse2 to adopt a SUMO-binding configuration and provide space for E2 and substrate interaction.

To address reviewer's comment that "we report a lot of moderate or mild effects, but do not offer definite results or conclusions", we have now highlighted fold changes amongst different conditions both in text and on plots. We also adjusted the plots to improve readability. Below, we present key fold changes that illustrate the strong convergence of *in vivo* and *in vitro* data in support of our conclusions.

***In vitro data***:

**1.** dsDNA and HJ DNA increased Smc5-Smc6 SUMOylation products up to 12-fold when Smc5/6 was used as E3, whereas a less than 2-fold increase was seen when Nse2-Smc5 was used as E3 (Fig. 2b, 2d).
**2.** HJ DNA increased Sgs1 SUMOylation products up to 3.8-fold when Smc5/6 was used as E3, whereas only 1.1-fold increase was seen when Nse2-Smc5 was used as E3 (Fig. 2e, 2f).
**3.** $Nse3^{DNAm}$ reduced DNA stimulatory effect up to 3.3-fold compared with the wild-type complex (Fig. 4b).
**4.** $Smc5/6^{EQ}$ that stabilizes the O-shaped conformation increased SUMOylation level up to 3.1-fold (Fig. 5c).
**5.** $Smc5/6^{KE}$ defective in ATP and DNA binding reduced Smc5-Smc6 SUMOylation up to 3.5-fold in the presence of DNA (Fig. 5a, 5b).

***Cellular data***:

**1.** DNA binding mutants of Smc5/6 rendered Smc5-Smc6 SUMOylation barely detectable (Figs. 6a-6d).
**2.** These mutants reduced Sgs1 SUMOylation up to 4-fold (Fig. 6e).
**3.** $Smc5^{EQ}$ increased the percentage of SUMOylated Smc5 up to 13-fold (Fig. 7c).
**4.** $Smc5^{KE}$ reduced Smc5 SUMOylation to background level and Sgs1 SUMOylation 2-fold (Fig. 7a,7e).

**MAJOR POINTS**

**Page 4**, lines 139-144 The authors report that acute depletion of Smc6, Nse4 or Nse6 leads to a reduction in SUMOylation of Smc5 - and that depletion of Smc5 (and thus Nse2), Nse4, and Nse6 lead to a reduction in SUMOylation of Smc6. Given the interdependence of the complex on each of its constituent components, the only real insight offered here, is the potential effect of depleting Nse6 on the SUMOylation pathway [but see other comments below].
Thanks for acknowledging the new insight offered from this result. As these results are not

central for our main conclusions, we have now moved them from Figure 1 & Suppl Figure 1 to Suppl. Figure 6 to avoid distraction from findings that are more pertinent to the main conclusions.

**Page 5,** lines 197-204 + Page 6, lines 205-211 “This is exemplified by Sgs1, the SUMOylation of which is enhanced by its association with DNA repair intermediates” “These observations let us ask whether non-SUMO E3 subunits of Smc5/6 can contribute to SUMOylation in the presence of DNA” “Smc5/6 can facilitate HJ processing partly by promoting Sgs1 SUMOylation and preferentially binds to ds-ssDNA junctions and supercoiled DNA over dsDNA and ssDNA”. Given the statements above and the cited references, it is not clear what insight the authors are providing, over and above what has already been published. Furthermore, it is already known that the SUMOylation activity of Nse2 is stimulated by the presence of single-stranded DNA [Varejão et al, EMBO J, 2018, Varejão et al, Nature Comms 2021]. How do the authors square their observations with the published literature?

As detailed above, our study represents the first biochemical investigation of the complete Smc5/6 complex in SUMOylation and provides several new insights into its functional mechanisms. To avoid redundancy, we highlight only two of the several new insights provided by our work that are the most pertinent to the cited two references. First, while the two cited papers showed that ssDNA is superior to dsDNA in stimulating Nse2-Smc5-based SUMOylation, we found the opposite for Smc5/6. Second, while Smc5-arm residues were found to support ssDNA-based stimulation of Nse2-Smc5 activity, we showed that dsDNA binding sites on Smc5-6 head and Nse3-4 contribute to dsDNA-based stimulation, a finding in line with structural and single molecule data regarding how Smc5/6 interacts with DNA [18-25]. These and other insights as described above highlight new perspectives gained from studying the complete Smc5/6 complex, over those from examining just Nse2 and Smc5, in SUMOylation.

Our data also offer mechanistic explanation for previous findings cited in reviewer’s comments. For example, our data provide an explanation of how Sgs1 SUMOylation by Smc5/6 can be enhanced by repair intermediates, as we show that DNA supports substrate and enzyme proximity in this process. We have now better described our novel findings and compared them with published data (Page 10, lines 441-446; Page 11, lines 465-466).

The authors also do not measure any kinetics for their reconstituted SUMOylation assays, making it difficult to accurately compare the activity of the different reconstituted complexes. This is especially apparent when comparing the 6-subunit Smc5/6 complex to the 8-subunit complex (i.e. +/- Nse5/6), for example in Figure 2 at the 4-minute time point, there is no statistical difference between the two complexes; closer observation shows that a single outlier in both datasets serves to create the visual difference.

All biochemical tests in this work were conducted at two to three time points to provide a dynamic view of the reactions, and results are quantified to assess SUMOylation efficiencies under different conditions. Such data are sufficient to inform the mechanisms of Smc5/6-mediated SUMOylation. For example, Figure 1 shows that the core- or holo- Smc5/6 did not differ at 2 or 4 min into the reactions for producing SUMOylated Smc5-Smc6 or Sgs1. These data suggest that the two forms of Smc5/6 have a similar capacity in SUMOylation under the same experimental conditions. We were careful to use multiple repeats and statistical analyses to guide our conclusions.

Our work aims to better understand physiologically relevant SUMOylation carried out by the complete Smc5/6 complexes (~500 kDa) for self-SUMOylation and SUMOylation of the full-length Sgs1 with its partner proteins (~280 kDa). These large complexes are difficult to purify, and their low concentrations prevent detailed kinetic analyses. We have noted these technical

limitations (Page 14, lines 649-651); however, such limitations do not prevent us from reaching main conclusions listed above. For example, our data strongly support dsDNA- and ATP-binding by multiple subunits of Smc5/6 regulate its SUMOylation activity. Future improvements of protein purification can make detailed kinetic studies feasible.

**Page 6**, lines 213-244 “We added each type of DNA at the same nucleotide concentration in SUMOylation reactions containing core-Smc5/6” The importance of determining kinetics is once again seen in the experiments presented in Figure 3; here the differences observed after 10 minutes incubation time between the different subunits could be considered negligible; all supplied substrates are stimulatory with respect to the “no DNA” control. Also, it is imperative that experiments are carried out in conditions close to, or in excess of the Kd for the interaction of the complex with the provided substrate, to facilitate direct comparison.

Quantification of our data at different time points support the conclusion that the five types of DNA examined enhanced Smc5/6-mediated SUMOylation. However, dsDNA is superior to ssDNA in stimulation, since ssDNA did not show stimulation at 5-min timepoint whereas dsDNA exhibited 10-fold stimulation (Figure 2c). As described above, technical limitations prevent more detailed kinetic analyses to further dissect the differences of all types of DNA, which is not the focus of this work. We note that Smc5/6 interacts with dsDNA and ssDNA at a Kd of approximately 100 nM[23], which is close to the DNA concentration used in most of the SUMOylation reactions. We have modified the text to make this clearer (Page 14, lines 634-636).

**Page 6**, Lines 230-234 “In the presence of core-Smc5/6, HJ and ds-ssDNA led to increased Sgs1 SUMOylation compared with no DNA control” Presumably, Sgs1 is active in this assay? The authors should understand the contribution of the helicase’s activity to the stimulation of SUMOylation, i.e. generation of a more favourable DNA substrate upon unwinding.

To prevent interference from DNA unwinding by Sgs1, SUMOylation reactions included non-migratable HJ DNA, which can be processed by Sgs1 only in the presence of RPA[26-28]. We confirmed this by showing that under the SUMOylation reaction conditions, HJ DNA and ds-ssDNA remain intact (new Suppl Fig. 2a). We have also included new data to show that our Sgs1 preparation was active as it unwound ds-ssDNA in the presence of yeast RPA (new Suppl. Fig. 2b).

**Page 6**, lines 253-254 “A recent cryo-EM structure showed that a single core-Smc5/6 binds to 20-bp dsDNA. Thus, the minimal DNA length required for SUMOylation stimulation raised the possibility that two core-Smc5/6 complexes may sumoylate each other in-trans, with one acting as the E3 and the other as the substrate, when both are bound to the same dsDNA molecule” Others have shown that more than one Smc5/6 complex can bind to longer stretches of dsDNA, and that the loop extrusion behaviour of the complex requires a dimer; please take care to provide citations to previous work that provide the foundation to your study.

We have included citations of the study showing dimer Smc5/6 can perform DNA loop extrusion (Page 6, line 227).

**Page 8**, line 305-212 “In each case, levels of SUMOylated Smc5-Smc6 were moderately reduced compared with the wild-type core-Smc5/6 (Fig. 5b), as were SUMOylated Sgs1 levels (Fig. 5c). The moderate effects seen for each mutant are likely because DNA binding sites on the other subunits enable partial DNA association. These results provide in vitro evidence that DNA binding via four non-SUMO E3 subunits contributes to DNA enhanced Smc5/6 SUMO E3

activity". The authors shy away from the more obvious conclusion that the complexes containing mutations which affect dsDNA binding have very little effect on the SUMOylation activity of the Smc5/6 complex. They should test the effects of hinge mutations that disrupt the ability of the complex to interact with single-stranded DNA; this is especially important given the authors previous statement that Smc5/6 "preferentially binds to ds-ssDNA junctions"; and previous publications linking the SUMOylation activity of Nse2 to single-stranded DNA binding.

As described above, we found that dsDNA stimulates Smc5/6's SUMOylation activity more effectively than ssDNA, in contrast to conclusions drawn from studies of the Nse2-Smc5 dimer alone. Building onto this finding, our subsequent investigation focused on how dsDNA and dsDNA-containing HJ structures confer stimulation. While hinge binding to ssDNA is not relevant to this investigation, we have now discussed its potential involvement in SUMOylation (Page 11; Lines 465-466).

Our mutational analyses further suggest that dsDNA stimulates Smc5/6's SUMOylation activity via binding to four of its subunits. We showed that compared with wild-type Smc5/6, four Smc5/6 variants, each containing mutations on structurally mapped dsDNA-binding residues on a distinct subunit, reduced SUMOylation in the presence of HJ DNA. We now depict the fold changes caused by the mutants on the plots and highlighted that they caused up to 3.3-fold reduction in Smc5-Smc6 SUMOylation and 2.7-fold reduction in Sgs1 SUMOylation compared with the wild-type complex (Figs 4b-4c). These changes are both significant and consistent with *in vivo* data provided (Figure 6).

Furthermore, given that Nse2 serves to bridge the two arms of the Smc5/6 complex (see Hallett et al, CryoEM structure of the Smc5/6 holo-complex, 2002). The authors should consider that ATP-binding serves to disrupt this interface (I to O transition), allowing access of the Nse2 subunit to DNA substrates (ssDNA, ds-ssDNA) leading to stimulation of its activity.
We have already included this view in the Discussion and have now added the said publication (Page 11, lines 485-488).

Figure 3 Could the authors explain why there are more replicates for experiments with the Holliday Junction substrate. Ideally all experiments should have the same number, in order not to affect the numerical results of a two-tailed unpaired t-test; consider use of a Welch t-test, when comparing data with unequal sample sizes / variances.
As HJ structures are the most biologically relevant form of DNA for Sgs1 SUMOylation based on previous studies[3,4,29], more tests were done using HJ structures. We took the reviewer's suggestion and included Welsh t-test results when comparing data with unequal sample sizes and variants. Importantly, the new analyses did not change our conclusions.

MINOR

Consider altering SUMOylation to SUMOylation throughout. The suggested change is made.

Given that Nse5/6 are not obligatory parts of the Smc5/6 complex in either S. pombe or in humans, the use of the term 'holo-complex' should be ideally be reserved for the 6-subunit complex, and not used for the 8-subunit complex.
We have modified the text to be clearer that this work examines the budding yeast Smc5/6 that has been shown to contain eight subunits by several studies (Page 3, lines 70-73). For example, affinity-based purification of the endogenous Nse2 (aka Mms21) recovered all eight subunits of the complex including Nse5-6[1], and the eight-subunit complex was purified as one entity from endogenous sources by pulling down Smc5[30]. More recently, several labs have

demonstrated the biochemical integrity of the eight-subunit budding yeast Smc5/6 complex and that all subunits were co-purified in stoichiometric amounts and remained associated with each other as a complex even in 500 mM salt conditions[18-21,24,31,32]. A cryo-EM structure further revealed the eight subunits show extensive interaction with each other forming the holo-complex[13].

The use of 'non-SUMO E3' subunits as a terminology is potentially confusing, especially as there is actually only one SUMO E3 subunit, i.e. Nse2. We have now stated that "non-SUMO E3 subunits" or "non-E3 subunits" include Smc5, Smc6, Nse1, and Nse3-6 (Page 3, line 78).

Page 6, lines 236-244 "Significantly, the additional of holo-Smc5/6 led to poly- and multi-SUMOylation species of Sgs1, the level of which was increased by inclusion of HJ DNA in the reactions". How are these being enumerated? Can the authors distinguish between the different species?
Poly- and multi-SUMOylation species of Sgs1 forms are difficult to be separated by SDS-PAGE due to the large size of Sgs1, which migrates close to 250 kDa on gels. We clarified in the text that these forms are collectively referred to as poly- and multi-SUMOylated Sgs1 forms (Page 21, lines 936-938).

**References**


1. Zhao, X. & Blobel, G. A SUMO ligase is part of a nuclear multiprotein complex that affects DNA repair and chromosomal organization. *Proc Natl Acad Sci U S A* **102**, 4777-4782 (2005).
2. Bermudez-Lopez, M. et al. ATPase-dependent control of the Mms21 SUMO ligase during DNA repair. *PLoS Biol* **13**, e1002089 (2015).
3. Bonner, J.N. et al. Smc5/6 mediated sumoylation of the Sgs1-Top3-Rmi1 complex promotes removal of recombination intermediates. *Cell Rep* **16**, 368-378 (2016).
4. Bermudez-Lopez, M. et al. Sgs1's roles in DNA end resection, HJ dissolution, and crossover suppression require a two-step SUMO regulation dependent on Smc5/6. *Genes Dev* **30**, 1339-1356 (2016).
5. Van Hooff, J.J.E. et al. Repeated duplications and losses shaped SMC complex evolution from archaeal ancestors to modern eukaryotes. *Cell Rep* **44**, 115855 (2025).
6. Potts, P.R. & Yu, H. Human MMS21/NSE2 is a SUMO ligase required for DNA repair. *Mol Cell Biol* **25**, 7021-7032 (2005).
7. Andrews, E.A. et al. Nse2, a component of the Smc5-6 complex, is a SUMO ligase required for the response to DNA damage. *Mol Cell Biol* **25**, 185-196 (2005).
8. Peng, X.P. & Zhao, X. The multi-functional Smc5/6 complex in genome protection and disease. *Nat Struct Mol Biol* **30**, 724-734 (2023).
9. Roy, S., Adhikary, H. & D'Amours, D. The SMC5/6 complex: folding chromosomes back into shape when genomes take a break. *Nucleic Acids Res* **52**, 2112-2129 (2024).
10. Hopf, L.V.M. et al. Structure of CRL7(FBXW8) reveals coupling with CUL1-RBX1/ROC1 for multi-cullin-RING E3-catalyzed ubiquitin ligation. *Nat Struct Mol Biol* **29**, 854-862 (2022).
11. Alfieri, C. et al. Molecular basis of APC/C regulation by the spindle assembly checkpoint. *Nature* **536**, 431-436 (2016).
12. Zhang, S. et al. Molecular mechanism of APC/C activation by mitotic phosphorylation. *Nature* **533**, 260-264 (2016).
13. Li, Q. et al. Cryo-EM structures of Smc5/6 in multiple states reveal its assembly and functional mechanisms. *Nat Struct Mol Biol* **31**, 1532-1542 (2024).
14. Varejao, N. et al. DNA activates the Nse2/Mms21 SUMO E3 ligase in the Smc5/6 complex. *EMBO J* **37**, e98306 (2018).
15. Varejao, N. et al. Structural basis for the E3 ligase activity enhancement of yeast Nse2 by SUMO-interacting motifs. *Nat Commun* **12**, 7013 (2021).
16. Takahashi, Y. et al. Cooperation of sumoylated chromosomal proteins in rDNA maintenance. *PLoS Genet* **4**, e1000215 (2008).

17. Zapatka, M. et al. Sumoylation of Smc5 promotes error-free bypass at damaged replication forks. *Cell Rep* **29**, 3160-3172 e4 (2019).
18. Chang, J.T. et al. Smc5/6's multifaceted DNA binding capacities stabilize branched DNA structures. *Nat Commun* **13**, 7179 (2022).
19. Tanasie, N.L. et al. Stabilization of DNA fork junctions by Smc5/6 complexes revealed by single-molecule imaging. *Cell Rep* **41**, 111778 (2022).
20. Pradhan, B. et al. The Smc5/6 complex is a DNA loop-extruding motor. *Nature* **616**, 843-848 (2023).
21. Gutierrez-Escribano, P. et al. Purified Smc5/6 complex exhibits DNA substrate recognition and compaction. *Mol Cell* **80**, 1039-1054 e6 (2020).
22. Hallett, S.T. et al. Cryo-EM structure of the Smc5/6 holo-complex. *Nucleic Acids Res* **50**, 9505-9520 (2022).
23. Taschner, M. et al. Nse5/6 inhibits the Smc5/6 ATPase and modulates DNA substrate binding. *EMBO J* **40**, e107807 (2021).
24. Taschner, M. & Gruber, S. DNA segment capture by Smc5/6 holocomplexes. *Nat Struct Mol Biol* **30**, 619-628 (2023).
25. Yu, Y. et al. Cryo-EM structure of DNA-bound Smc5/6 reveals DNA clamping enabled by multi-subunit conformational changes. *Proc Natl Acad Sci U S A* **119**, e2202799119 (2022).
26. Wang, W. et al. A DNA nick at Ku-blocked double-strand break ends serves as an entry site for exonuclease 1 (Exo1) or Sgs1-Dna2 in long-range DNA end resection. *J Biol Chem* **293**, 17061-17069 (2018).
27. Cejka, P. & Kowalczykowski, S.C. The full-length Saccharomyces cerevisiae Sgs1 protein is a vigorous DNA helicase that preferentially unwinds holliday junctions. *J Biol Chem* **285**, 8290-301 (2010).
28. Cejka, P. et al. DNA end resection by Dna2-Sgs1-RPA and its stimulation by Top3-Rmi1 and Mre11-Rad50-Xrs2. *Nature* **467**, 112-6 (2010).
29. Li, S. et al. Multifaceted regulation of the sumoylation of the Sgs1 DNA helicase. *J Biol Chem* **298**, 102092 (2022).
30. Kanno, T., Berta, D.G. & Sjogren, C. The Smc5/6 complex is an ATP-dependent intermolecular DNA linker. *Cell Rep* **12**, 1471-1482 (2015).
31. Li, S. et al. Molecular basis for Nse5-6 mediated regulation of Smc5/6 functions. *Proc Natl Acad Sci U S A* **120**, e2310924120 (2023).
32. Yu, Y. et al. Integrative analysis reveals unique structural and functional features of the Smc5/6 complex. *Proc Natl Acad Sci U S A* **118**, e2026844118 (2021).

We thank both reviewers for evaluating our manuscript. We have now addressed the reviewers' points in a further revised manuscript. Our detailed responses to each comment (below) and textual changes are colored red.

**Reviewer #1**. The authors rewrote the article and significantly improved its quality. The revised version has a more logical sequence of results and clearer conclusions. I have only a minor objection to the arrangement of panels in the Figures – it would be clearer to arrange panels A - B ... horizontally (side by side, not one above the other and then randomly below).

Thank you for the positive feedback. We have re-ordered panels as suggested in the relevant figures.

**Reviewer #3**. The authors have included additional data, improved quantification, and expanded the discussion to address Reviewer #2's comments, and in doing so have strengthened both the clarity and overall presentation of the work. That said, several important concerns raised by Reviewer #2 remain only partially addressed. The main conclusions of the manuscript, that ATP binding and DNA stimulate Smc5/6-dependent SUMOylation, are already established in the literature. While the use of the full Smc5/6 complex provides a more physiological context and represents a clear technical advance, the conceptual novelty relative to previous studies remains somewhat limited. As it stands, the manuscript primarily refines existing models rather than extending significantly our understanding.

We appreciate the reviewer's positive comment that the multiple changes we incorporated have strengthened the work.

The conceptual novelty of this work goes beyond the aforementioned conclusion and includes important and novel mechanisms underlying ATP/DNA-based stimulation. The mechanisms revealed by using the entire Smc5/6 complex are in stark contrast with those proposed using only the Nse2-Smc5 dimer (Varejao et al. EMBO J, 2018; Bermudez-Lopez et al. PLoS Biol, 2015). These represent significant advances beyond a mere refinement of existing models, as detailed below.

First, we show that dsDNA stimulates SUMOylation by <u>enabling proximity</u> between the SUMO E3 and the substrate, and that DNA-binding sites located on the Smc5 and Smc6 head regions and Nse3-4 contribute to this stimulation. In contrast, Varejao et al. proposed that ssDNA stimulates SUMOylation by <u>changing the conformation</u> of the Nse2-Smc5 dimer, and that a DNA-binding site on the Smc5 arm region is responsible for the stimulation. These are fundamentally different mechanisms: our model invokes proximity-based stimulation through multiple DNA-binding sites distributed across the complex, whereas the previous model relies on conformational change within a partial subcomplex acting through a single DNA-binding site. Because the Nse2-Smc5 dimer does not exist as such in cells, the mechanism derived from our work using the full Smc5/6 complex represents the first physiologically relevant model.

Second, our data suggest that ATP stimulates SUMOylation by <u>enhancing Smc5/6-DNA interaction and inducing conformational change across the entire complex</u>. ATP is known to be sandwiched between the Smc5 and Smc6 head regions, as the ATP binding sites are composed of residues from both proteins. It is thus unlikely that ATP can productively bind to the Nse2-Smc5 dimer alone – a critical limitation of previous studies that relied on this dimer. Even if one were to ignore this issue, Bermudez-Lopez et al. proposed that ATP <u>compacts the Nse2-Smc5 dimer</u> to stimulate SUMOylation, whereas our data indicate that ATP acts by promoting DNA engagement and inducing conformational changes that span the entire complex. Our proposed mechanism for the full Smc5/6 complex thus differs fundamentally from that proposed for the Nse2-Smc5 dimer.

Third, we show that while Nse2-Smc5 exhibited higher basal activity than Smc5/6 in the absence of DNA, dsDNA conferred a 12-fold stimulation of SUMOylation when Smc5/6 was used as the E3, in contrast with less than 2-fold stimulation when Nse2-Smc5 was used as the E3. This result demonstrates that the intact Smc5/6 complex minimizes promiscuous SUMOylation in the absence of DNA while achieving much stronger activity in its presence. This is an important concept not previously proposed which provides a mechanistic basis for how SUMOylation specificity is achieved in vivo. Notably, this regulatory logic in which suppression of basal activity coupled with strong stimulus-dependent activation could only be revealed by studying the intact complex rather than the Nse2-Smc5 dimer alone.

In summary, our work puts forward new models and fresh perspectives for understanding how DNA and ATP stimulate Smc5/6-based SUMOylation. These advances were made possible by examining the full Smc5/6 complex, which not only provides a technical advance, as pointed out by the reviewer, but critically, enables the discovery of physiologically relevant mechanisms that were not previously known. We respectfully submit that these findings constitute substantial conceptual advances rather than incremental refinements of prior models. We have now edited the text to more clearly distinguish the models derived from our data from those derived from studying the Nse2-Smc5 dimer (page 12, lines 523-527).

The most potentially novel aspect of the study is the reported difference in DNA substrate preference, namely that double-stranded DNA (dsDNA) appears more stimulatory than single-stranded DNA (ssDNA), this is in contrast to earlier work using the Nse2–Smc5 subcomplex. However, this important observation is not yet developed with sufficient depth. In this context, a key request from Reviewer #2, arguably the most important one, remains unaddressed. The reviewer asked testing hinge mutants that disrupt ssDNA binding in the SUMOylation assays. Given the well-established role of the Smc5/6 hinge in mediating ssDNA interactions, this experiment is essential to directly compare how dsDNA vs ssDNA binding contributes to SUMOylation activity in the context of the full complex. Importantly, this analysis should not be limited to a single condition. A systematic comparison between hinge mutant and wild-type complexes across the full range of DNA substrates used in the study, including ssDNA, dsDNA, and DNA junctions, should be included.

We appreciate the reviewer's comment on one of our novel findings. However, we would like to also highlight our in-depth mechanistic studies (Figures 2d-2f; 3a-3c; 4a-4c; 6a-6e; S4b-S4c) that develop the initial findings, revealing how dsDNA achieves stimulation, as outlined above.

It is noteworthy that Varejao et al. (EMBO J, 2018; Figure 2B) showed that *the hinge is not required for DNA stimulation of SUMOylation* by Nse2-Smc5. Reviewer #2's original request to test a hinge mutant defective in ssDNA binding was based on the assertion that Smc5/6 complexes carrying dsDNA-binding site mutations "have very little effect on sumoylation." On the contrary, our data showed that these mutants led to more than 3-fold reduction of SUMOylation in vitro (Figures 4b-4c), a result that is further strengthened by consistent in vivo findings (Figures 6a-6e). We addressed this erroneous assessment in detail in our previous rebuttal letter.

We would also like to point out that the ssDNA-binding sites of the hinge region have not been structurally mapped. Two reports have suggested different sets of residues on the hinge regions (Alt et al. 2017, PMID 28134253; Liu et al. 2025, PMID 41290596), and these differ from predictions based on AlphaFold models (data not shown). As such, substantial work would be needed to identify the bona fide ssDNA-binding residues of the Smc5 and Smc6 hinge regions before a proper mutant could be generated that specifically perturbs this feature without disrupting other hinge structures and functions. This uncertainty makes it premature to interpret results from any currently available hinge mutant as specifically reflecting loss of ssDNA binding.

Finally, we showed that a 80-nt ssDNA did not stimulate Smc5/6-based SUMOylation at 5 minutes, when dsDNA conferred >10-fold stimulation (Figure 2a-2b). This 80-nt ssDNA only showed stimulation at the 10-min timepoint (Figure 2a-2b). We suspect that this much-delayed effect may be related to a high probability of it forming dsDNA hairpins at several places, as predicted by the Mfold program (PMID: 12824337). To test this idea, we examined 80-nt poly-dT ssDNA, which does not form secondary DNA structures. We found that it showed no stimulation of SUMOylation at either 5 min or 10 min of the reactions, while the control dsDNA and ds-ssDNA did (Figure 1a-1b for reviewer). This finding strengthens our conclusion and indicates that studies of ssDNA-binding mutants would not provide further insight into DNA-based stimulation of SUMOylation by the Smc5/6 complex.

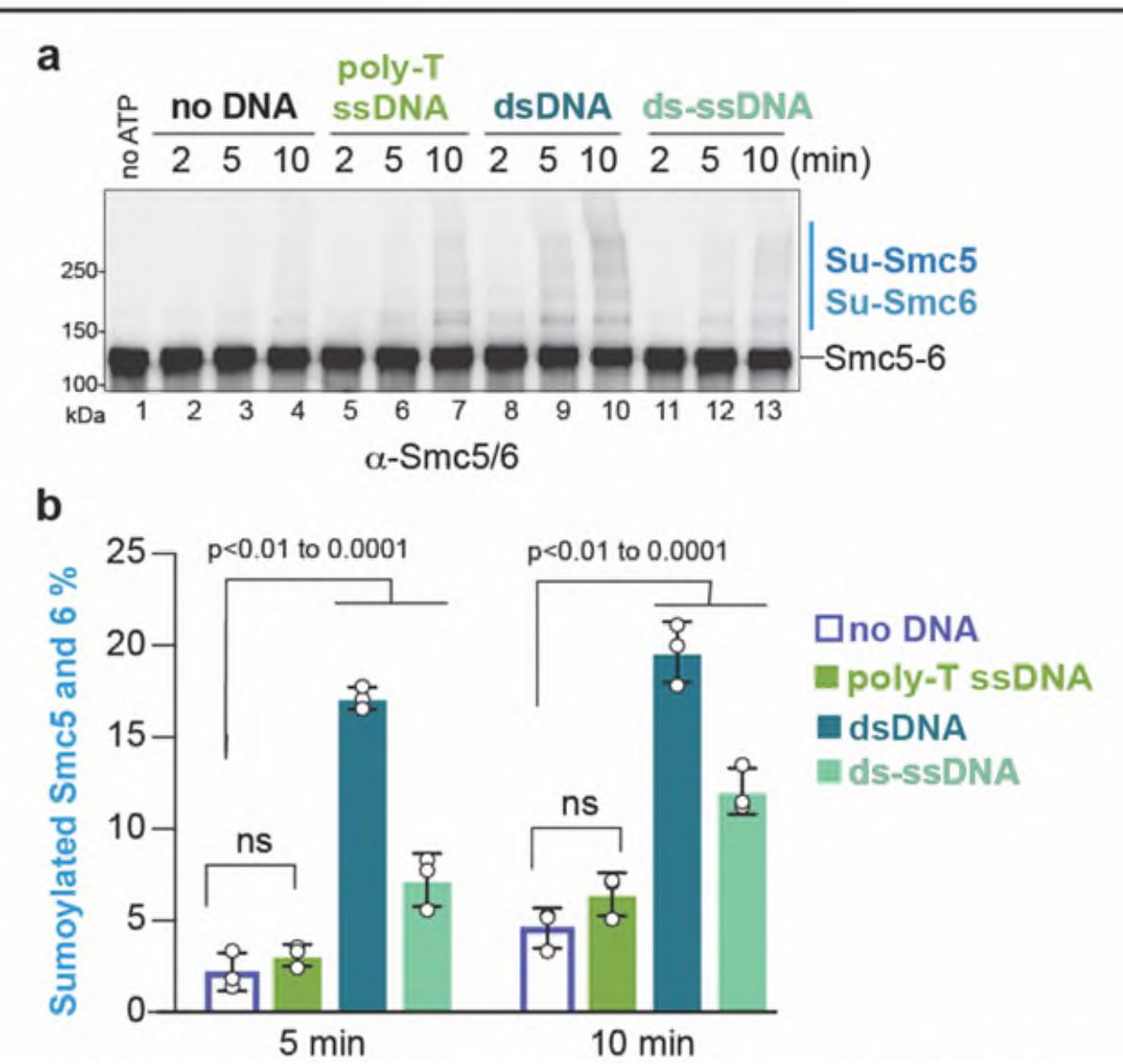

**Figure 1 for reviewers**. **a)** A representative blot of SUMOylation reactions comparing the effects of poly-dT ssDNA, dsDNA, and ds-ssDNA. The lenght of DNA is 80-nt or 80-bp. Reactions were performed as in Figure 2b. **b)** Percentage of SUMOylated Smc5-6. Quantified results from three independent experiments are plotted; means, SDs, and p-values (unpaired t-tests) indicated.

In summary, the manuscript is technically improved and clearer following revision. However, directly addressing the role of hinge-mediated ssDNA binding through systematic analysis of hinge mutants across relevant DNA substrates and their effect on SUMOylation is critical to elevate the conceptual impact of the work otherwise the novelty of the work remains rather limited over previous studies.

We have addressed the reviewer's comments in detail as described above. To summarize, both our data and published findings indicate that hinge-mediated DNA binding plays little role in Smc5/6-based SUMOylation. Specifically, Varejao et al. demonstrated that the hinge is dispensable for DNA-stimulated SUMOylation, and our poly-dT experiment demonstrates that ssDNA per se does not stimulate SUMOylation by the intact complex. Generating an Smc5/6 complex carrying mutations at the hinge regions that specifically abolish ssDNA binding without affecting other attributes of the complex, such as hinge dimerization, requires careful determination of the bona fide ssDNA-binding residues, which remain in dispute in the literature (see above). This undertaking is beyond the scope of this work. Furthermore, we respectfully disagree with the assessment that the novelty of our work is contingent upon this single experiment. We have outlined above the novel mechanisms and concepts underlying ATP- and DNA-based stimulation of SUMOylation that our work has revealed, which differ fundamentally from those proposed in previous studies using the Nse2-Smc5 dimer. These include (1) a proximity-based model for dsDNA stimulation involving multiple DNA-binding sites across the complex, (2) a mechanism for ATP stimulation involving enhanced DNA engagement and complex-wide conformational change, and (3) the discovery that the intact complex suppresses basal SUMOylation while achieving strong DNA-dependent activation—a regulatory logic that provides mechanistic insight into SUMOylation specificity in vivo.

**Reviewer #3**. I thank the authors for their thorough and detailed rebuttal. The additional poly-dT ssDNA experiment is informative and goes a meaningful way toward addressing the central concern about ssDNA versus dsDNA stimulation, demonstrating that structure-free ssDNA fails to stimulate SUMOylation by the intact complex is a substantive result that supports their conclusions. I am also persuaded by the mechanistic contrasts drawn with the Nse2-Smc5 dimer studies, particularly the proximity-based model, the ATP-DNA engagement argument, and the basal suppression concept. Taken together, these represent genuine conceptual advances and I am satisfied that the manuscript merits publication. That said, I maintain that the hinge mutant question has not been fully resolved, it has been sidestepped rather than answered. The argument that ssDNA-binding residues are structurally uncertain and that generating a clean mutant is beyond scope is not entirely satisfying. The poly-dT experiment provides indirect evidence, but a direct test of hinge-mediated ssDNA binding in the context of the full complex would have been the cleaner experiment. I would encourage the authors to acknowledge this limitation explicitly in the manuscript rather than presenting the ssDNA insensitivity conclusion with full confidence. I recommend acceptance subject to this minor textual revision.

We thank Reviewer #3 for evaluating our revised manuscript. We appreciate reviewer's positive comments and support of publication. We agree that a hinge DNA binding mutant complex will be interesting to test in the future and have acknowledged this limitation in Discussion (page 11, line 465-469; page12, line 525-530).
